# Low Serum Levels of Interleukin-6 (IL-6) and Monocyte Chemoattractant Protein-1 (MCP-1) in Immediate Postpartum Mexican Women With Gestational Diabetes

**DOI:** 10.7759/cureus.78647

**Published:** 2025-02-06

**Authors:** Mayra-Esther Rojas-Quintana, Karla-Maria Lopez-Martinez, Elizabeth Bautista-Rodriguez, Ricardo Marquez-Velasco, Sergio Cásarez-Alvarado, Fatima-Irais Sierra-Pineda, Jose Luis Cortez-Sanchez, Oscar Peralta-Zaragoza, Elie Girgis-Elkassis

**Affiliations:** 1 Department of Biotechnology, Universidad Popular Autónoma del Estado de Puebla, Puebla, MEX; 2 Faculty of Health Sciences, Universidad Autónoma de Tlaxcala, Tlaxcala, MEX; 3 Department of Immunology, Instituto Nacional de Cardiología Ignacio Chávez, Mexico City, MEX; 4 Department of Genetics, Puebla Children's Hospital and Women's Hospital, Ministry of Health, Puebla, MEX; 5 Faculty of Chemical and Biological Sciences, Universidad Autónoma de Campeche, Campeche, MEX; 6 Department of Chronic Infections and Cancer, Center for Research in Infectious Diseases, National Institute of Public Health, Cuernavaca, MEX

**Keywords:** gestational diabetes, il-16, il-18, mcp-1, postpartum

## Abstract

Gestational diabetes (GD) is a multifactorial disease involving hormonal, metabolic, and inflammatory factors, among which cytokines such as interleukin-6 (IL-6), interleukin-18 (IL-18), and monocyte chemoattractant protein-1 (MCP-1) play key roles in immune regulation and tissue repair during pregnancy and postpartum. The aim of this study was to compare serum IL-6, IL-18, and MCP-1 concentrations in postpartum women with and without GD and to analyze sociodemographic characteristics and pregnancy-related complications. A descriptive, prospective, cross-sectional study was conducted on 32 postpartum women (16 GD and 16 controls). Sociodemographic, clinical, and biochemical data were collected. Serum cytokine levels were measured using the bead-based LEGENDplex™ assay (Biolegend, San Diego, CA, USA). Statistical analysis was performed using GraphPad Prism v9.0 (Dotmatics, Boston, MA, USA) with significance at p < 0.05. Women with gestational diabetes showed significant differences in sociodemographic factors, including higher rates of smoking (5, 31.2%) and alcohol consumption (4, 25%). Pregnancy complications such as hypertension (2, 12.5%), premature rupture of membranes (2, 12.5%), and macrosomia (5, 31.2%) were more common in the gestational diabetes group. Serum IL-6 and MCP-1 levels were significantly reduced in the gestational diabetes group compared with the control group, while IL-18 levels were not significantly different. Postpartum women with gestational diabetes have a unique inflammatory profile characterized by reduced IL-6 and MCP-1 levels. Furthermore, differences in sociodemographic factors and increased pregnancy-related complications highlight the multifactorial nature of gestational diabetes. These findings emphasize the need for further research to understand its long-term clinical impact.

## Introduction

Gestational diabetes (GD) is defined as hyperglycemia diagnosed during pregnancy that is not clearly overt diabetes [1] or as carbohydrate intolerance first recognized during pregnancy, which may or may not resolve postpartum [2]. The global prevalence of GD ranges from 1% to 20%, with recent increases to rates between 8.9% and 53.4%. This disorder is more prevalent in African, Hispanic, Indian, and Asian women than in Caucasian women [3]. In Mexico, there is no official incidence rate, but the first report in 1988 indicated 4%, with subsequent studies showing an increase to over 30% by 2016 [4]. Women with gestational diabetes increase the incidence of suffering from hypertensive disorders during pregnancy, preeclampsia, eclampsia, preterm delivery, macrosomia, hyperinsulinemia, and long-term consequences that affect both mother and children, among which are metabolic diseases, diabetes mellitus (mainly type 2), and obesity [5].

The GDM involves a complex interplay of hormonal, metabolic, and inflammatory factors. Hormonal changes during pregnancy, including elevated levels of placental lactogen and estrogens, contribute significantly to insulin resistance. Furthermore, altered glucose transporter type 4 (GLUT4) transporter function and enhanced lipolysis lead to increased free fatty acid levels, which interfere with glucose uptake and promote hepatic gluconeogenesis. Collectively, these processes, along with inflammation, profoundly affect maternal and neonatal health outcomes.

Inflammatory cytokines play a pivotal role in the development and progression of GD. Among these, interleukin-6 (IL-6), interleukin-18 (IL-18), and monocyte chemoattractant protein-1 (MCP-1) have garnered considerable attention due to their involvement in glucose metabolism, insulin signaling, and systemic inflammation. During pregnancy, inflammation is tightly regulated to support fetal development. However, a heightened inflammatory response at the end of the third trimester facilitates labor. IL-6 levels rise, promoting the synthesis of prostaglandins essential for uterine contractions. Simultaneously, IL-18 and MCP-1 are involved in the activation and infiltration of macrophages and natural killer (NK) cells into the myometrium and cervix, promoting cervical dilation and the initiation of labor [6].

In the postpartum period, inflammatory mechanisms shift to tissue repair and uterine homeostasis restoration. Cytokines such as IL-6 and tumor necrosis factor-alpha (TNF-α) contribute to wound healing and the prevention of complications such as hemorrhage or uterine and systemic infections. The objective of this study was to compare serum concentrations of IL-6, IL-18, and MCP-1 in puerperal women with and without GD and to report associated maternal complications. These findings aim to deepen the understanding of the inflammatory processes underlying GD and its impact on maternal health during the critical postpartum period.

## Materials and methods

This study employed a descriptive, cross-sectional, prospective, and observational design. We recruited participants from the puerperal women receiving care at the Hospital de la Mujer del Estado de Puebla (Ethics Committee Approval: HMN-013-2021). The sample size was determined by convenience, considering selection criteria between January 31, 2022, and August 29, 2022. A total of 32 patients were included, divided into two groups: the control group and the gestational diabetes (GD) group.

Control group

Sixteen puerperal women, aged 18-45 years, with normal pregnancies. Inclusion criteria for this group included normal, evolutionary pregnancies, with or without lung maturation therapy, access to prenatal care records, voluntary participation with signed informed consent, and being primigravid or multigravid with singleton pregnancies. Delivery methods included eutocic deliveries or non-elective cesarean sections without unstable fetal status. Participants were clinically healthy.

Gestational diabetes (GD) group

Sixteen puerperal women, aged 18-45 years, with a confirmed diagnosis of gestational diabetes (GD) through laboratory testing. Similar to the control group, these women may or may not have received lung maturation therapy, voluntarily participated, signed informed consent, and had singleton live births. Prenatal care was conducted within the participating health institution, with access to clinical records ensured.

Exclusion criteria

Women who did not consent to participate or donate samples, as well as those with the following conditions, were excluded: Rh sensitization, illicit substance use (e.g., drugs, tobacco, alcohol), severe anemia, cardiovascular diseases, fetal malformations, asthma, hemoglobinopathies, use of immunosuppressive drugs, infectious diseases, endocrinopathies (excluding diabetes mellitus), chronic communicable diseases, multiple pregnancies, adolescent pregnancies, and those who did not complete the study due to lack of periodic consultation or discontinuation of interventions.

Procedures

Upon enrollment, participants provided informed consent. Clinical and sociodemographic data were collected using a structured data collection sheet. Peripheral venous blood samples were obtained using the vacutainer system with ethylenediamine tetraacetic acid (EDTA) tubes. Blood samples were centrifuged at 3000 rpm to separate serum, which was analyzed for MCP-1, IL-6, and IL-18 levels using the LEGENDplex™ assay (Biolegend, San Diego, CA, USA, Cat: 740795) following the manufacturer’s instructions.

Flow cytometry protocol

Cytokine concentrations in serum were quantified using the LEGENDplex™ (Biolegend, San Diego, CA, USA, Cat: 740795) bead-based assay for MCP-1 (CCL2), IL-6, and IL-18. Serum samples were diluted 1:2 in the assay buffer. A 50 µL aliquot of the diluted sample or standard was added to each well of a V-bottom plate, followed by 25 µL of pre-vortexed mixed beads. The plate was incubated for two hours and centrifuged at 250 rpm for five minutes. Wells were washed twice with 200 µL of wash buffer. Detection antibodies (25 µL) were added and incubated, followed by 25 µL of streptavidin-phycoerythrin (SA-PE) without a washing step. After a 30-minute incubation, the plate underwent centrifugation and washing steps, and 150 µL of wash buffer was added to each well. Samples were transferred to microtubes for acquisition using a BD FACSAria™ Fusion (BD Biosciences, San Jose, CA, USA). Cytokine concentrations were calculated using LEGENDplex™ Data Analysis Software (Biolegend, San Diego, CA, USA), adhering strictly to the manufacturer’s protocol.

Statistical methods

Descriptive statistics were used to summarize the sociodemographic and clinical characteristics of the study population. Continuous variables, such as age and serum cytokine levels, were expressed as means and standard deviations. Categorical variables, including marital status, education level, and pregnancy complications, were presented as frequencies and percentages, and statistical analysis was performed using Fisher’s exact test. Comparative analyses between the control and GD groups were performed using a Student's t-test for continuous variables to evaluate differences in cytokine concentrations (MCP-1, IL-6, and IL-18). Statistical significance was set at p < 0.05. All statistical analyses were conducted using GraphPad Prism v9.0 software (Dotmatics, Boston, MA, USA).

## Results

Sociodemographic data

Table 1 presents the sociodemographic characteristics of the participants. The mean age of patients in the control group was 28 years, while in the gestational diabetes (GD) group it was 29 years. Both groups had an average of two pregnancies at the time of the study. Regarding marital status, 11 (68.7%) participants in both groups were in a common-law union. Additionally, four (25%) of the control group and three (18.7%) of the GD group were married, while one (6.2%) and two (12.5%), respectively, were single.

**Table 1:** Descriptive variables of mothers divided by study group (n=32). GD: gestational Diabetes; SD: standard deviation; min: minimum; max: maximum

Variable	Control group	GD group	p-value
Pregnancies, mean (min-max)	2 (1-4)	2 (1-4)	>0.9999
Age (Years), mean±SD (min-max)	28±5 (21-38)	29±7 (20-44)	0.4347
Marital status, n (%)			
Single	1 (6.2%)	2 (12.5%)	<0.9999
Married	4 (25%)	3 (18.7%)	<0.9999
Common-law union	11 (68.7%)	11 (68.7%)	>0.9999
Education level, n (%)			
None	0	2 (12.5%)	0.4839
Primary school	2 (6.2%)	4 (25%)	0.6539
Middle school	5 (31.2%)	4 (25%)	>0.9999
High school	6 (37.5%)	4 (25%)	0.7043
Bachelor’s degree/technical school	3 (18.7%)	2 (12.5%)	>0.999
Tobacco use, n (%)			
Yes	0	5 (31.2%)	0.0411*
No	11 (68.7%)	8 (50%)	
Alcoholism, n (%)			
Yes	0	4 (25%)	0.0983
No	11 (68.7%)	9 (56.2%)	

Educational attainment varied slightly between groups. Among women in the GD group, two (12.5%) had no formal education, compared to none in the control group. Furthermore, two (6.2%) of the control group completed primary school, and four (25%) of the GD group completed primary, middle, and high school. Notably, five women (31.2%) in the GD group reported a history of smoking, and four (25%) reported current or previous alcohol consumption. In contrast, none of the women in the control group reported these behaviors.

Complications during pregnancy 

Table 2 summarizes the pregnancy-related complications observed in the study groups. Diabetes mellitus (DM) and systemic arterial hypertension were exclusive to the GD group. Other complications were identified at lower frequencies. For instance, preeclampsia and urinary tract infections were present in both groups, three participants (18.7%) and one (6.2%), respectively. Premature rupture of membranes occurred in two (12.5%) cases in the GD group, while macrosomia was observed in five women (31.2%) of this group. The only complication observed in the control group was oligohydramnios in one woman (6.2%) of the group. The average weight at birth in the GD group was 3539 gr, while in the control group, the average weight was 2989 gr. In addition, the average gestational age was 36 weeks in the diabetes group and 39 weeks in the control group.

**Table 2:** Variables related to GD in the two groups of study (n=32). GD: gestational diabetes; gr: grams

Variable	Control group	GD group	p-value
	n (%)	n (%)	
Diabetes mellitus	0	2	0.0434*
Systemic arterial hypertension	0	2 (12.5%)	0.4839
Complications during pregnancy (excluding GD)	6 (37.5%)	7 (43.7%)	>0.9999
Preeclampsia	3 (18.7%)	3 (18.7%)	>0.9999
Urinary tract infections	1 (6.2%)	1 (6.2%)	>0.9999
Preterm rupture of membranes	0	2 (12.5%)	0.4839
Macrosomia	0	5 (31.2%)	0.0434*
Oligohydramnios	1 (6.2%)	0	>0.9999
Weight at birth (gr), mean±SD (min-max)	2989±374 (2190-3600)	3539±728 (2050-4650)	0.0117*
Gestational age (weeks of gestation), mean±SD (min-max)	39±1 (36-41)	36±1 (32-40)	0.0002*

Serum levels of IL-6, IL-18, and MCP-1 

Gestational diabetes is characterized as a condition involving low-grade inflammation. Accordingly, IL-6, IL-18, and MCP-1 were selected as inflammatory biomarkers, given their potential alterations during gestational diabetes.

**Figure 1:**
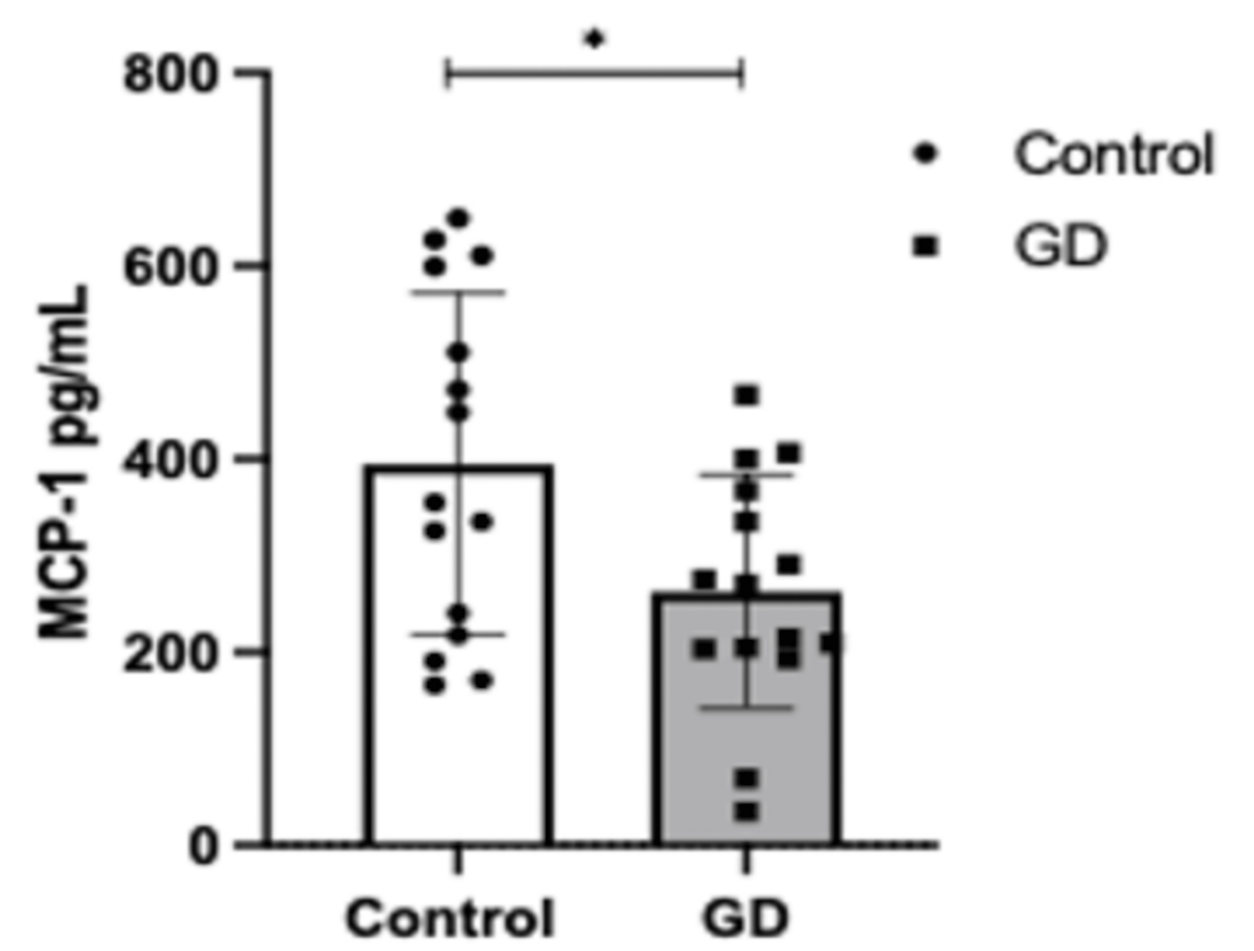
Serum levels of MCP-1 (pg/mL) in controls (n=16) vs. GD (n=16). Error bars represent ± standard deviation (SD). Means were compared using one-sample Student's t-test ∗p < 0.05. GD: gestational diabetes group; MCP-1:  monocyte chemoattractant protein 1

**Figure 2:**
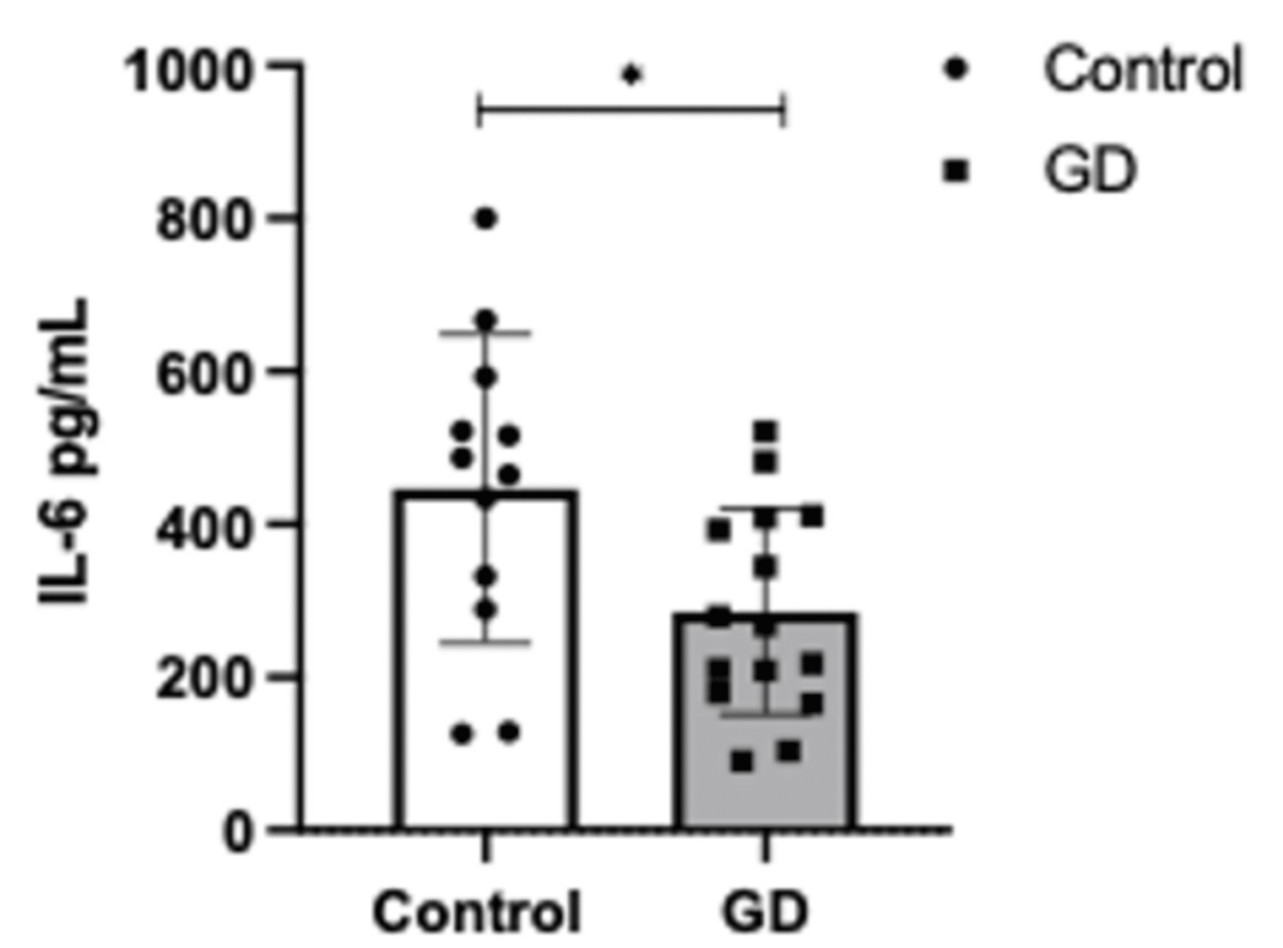
Serum levels of IL-6 (pg/mL) in controls (n=16) vs. GD (n=16). Error bars represent ± standard deviation (SD). Means were compared using one-sample Student's t-test ∗p < 0.05. GD: gestational diabetes group; Il-6: interleukin 6

**Figure 3:**
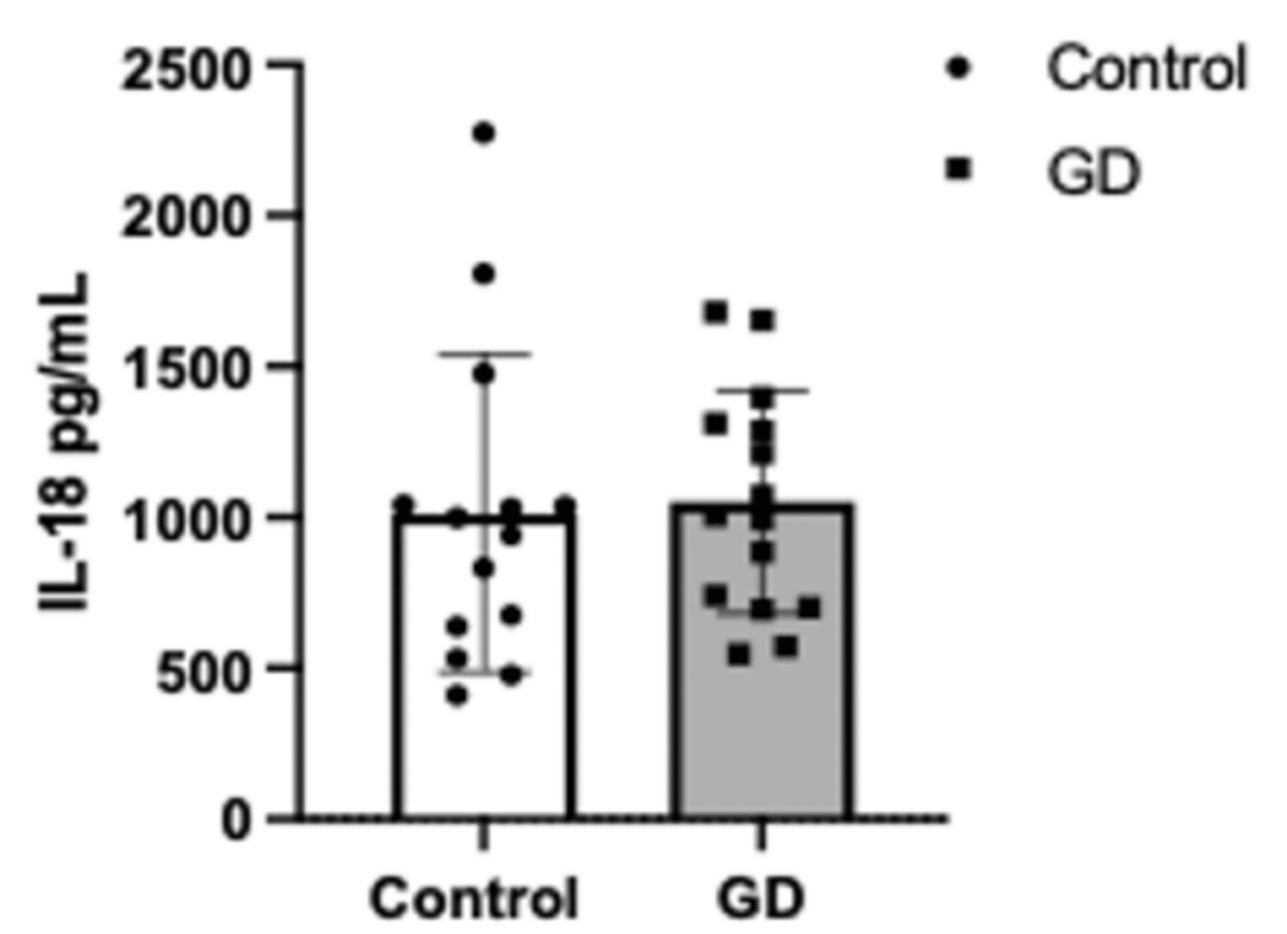
Serum levels of IL-18 (pg/mL) in controls (n=16) vs. GD (n=16) (p > 0.05). Error bars represent ± standard deviation (SD). Means were compared using one-sample Student’s t-test. GD: gestational diabetes group; IL-18: interleukin 18

A Student’s t-test was performed to assess differences in cytokine concentrations between the GD and control groups. The analysis revealed significantly higher levels of MCP-1 in the control group compared to the GD group (p = 0.0242, Figure 1). Similarly, IL-6 concentrations were significantly elevated in the control group (p = 0.0204, Figure 2). In contrast, no statistically significant differences were observed in IL-18 levels between the groups (Figure 3).

These findings suggest a distinct inflammatory profile in gestational diabetes, characterized by lower MCP-1 and IL-6 levels compared to women without the condition. Further research is warranted to explore the mechanistic underpinnings of these observations.

## Discussion

Gestational diabetes mellitus is a multifactorial condition associated with pathways that relate closely to both metabolic and inflammatory. This study describes sociodemographic factors, pregnancy outcomes, and inflammatory profiles of women with and without GD. These results will be important for understanding the profile of inflammation in GD, especially regarding cytokines such as IL-6, IL-18, and MCP-1.

Sociodemographic data revealed that women in the GD group shared similar marital and reproductive histories with the control group. However, differences in education and lifestyle behaviors, such as smoking and alcohol consumption, were notable. Specifically, five (31.2%) of the GD group reported a history of pregestational smoking, and four (25%) reported alcohol consumption, behaviors absent in the control group. These findings align with existing literature indicating that pregestational smoking or passive smoking during pregnancy is associated with an increased risk of GD [7,8]. While alcohol consumption has not been reported as a risk factor for GD, it has been linked to other complications, such as miscarriage [9].

The GD group experienced complications such as hypertension, preeclampsia, urinary tract infections, macrosomia, and premature rupture of membranes. These findings corroborate the established relationship between GD and adverse obstetric outcomes, including macrosomia and preeclampsia [10,11]. The absence of these complications in the control group underscores the critical role of GD in pregnancy complications.

This study evaluated MCP-1, IL-6, and IL-18 levels, which are essential during pregnancy for immune response and homeostasis maintenance [12-14]. Although these cytokines have been previously reported during pregnancy and their association with complications, this study analyzed their levels in the immediate postpartum period in women with and without GD.

Lower IL-6 levels were found in the GD group compared to controls. These results contrast with findings by Morisset et al., who reported elevated IL-6 levels during pregnancy and postpartum in women with GD [15]. IL-6 is a pleiotropic cytokine involved in energy metabolism, immune regulation, and neuroinflammation. Increased IL-6 expression in the cervix, myometrium, choriodecidua, and amnion has been linked to neutrophil and macrophage infiltration into the cervix at the onset of labor [16,17]. Elevated IL-6 levels are also associated with complications such as preeclampsia, preterm birth, fetal growth restriction, and GD [18,19].

Interestingly, this study did not observe increased IL-6 levels in the postpartum period in women with GD, which raises attention as low IL-6 levels might indicate a diminished immune response, creating a favorable environment for intrauterine infections [20,21]. Furthermore, low IL-6 levels could impair endometrial repair, as IL-6 is involved in maintaining endometrial homeostasis by inhibiting the proliferation of endometrial stromal cells [22].

On the other hand, IL-18, part of the IL-1 superfamily, plays a crucial role in Th1/Th2 balance and immune modulation during pregnancy [23]. Despite its well-documented role in inflammatory processes, no significant differences in IL-18 levels were observed between the GD and control groups in this study.

Our study also revealed significantly lower MCP-1 levels in the postpartum period in women with GD compared to controls, a finding that contrasts with existing literature suggesting increased MCP-1 expression in hyperglycemic and inflammatory states [24,25] and in women with GD [26]. MCP-1 regulates endometrial cell proliferation, differentiation, and apoptosis; promotes angiogenesis for adequate uteroplacental circulation; and ensures immune modulation for successful fetal development [14,27]. In the postpartum period, MCP-1 attracts monocytes and macrophages to remove necrotic tissue, promote endometrial healing, and prevent uterine infections. Dysregulation of MCP-1 could impair vascular remodeling and placental immune balance, exacerbating pregnancy complications [28]. Low MCP-1 levels might compromise these functions, as they have been reported in mothers with intrauterine growth restriction [14]. Furthermore, long-term studies are necessary, as low MCP-1 levels have been associated with major depressive disorder [29].

Strengths and limitations

This research was focused on the postpartum study of women subjected to gestational diabetes to offer valuable information regarding risk behavior, such as smoking and alcohol consumption, and obstetric complications in these females. The study is of the Mexican population, with a high prevalence of diabetes. The present study provides valuable data on inflammatory profiles (MCP-1, IL-6, and IL-18) during the postpartum period of women with GD. According to this discussion, even though striking relationships are proposed with low MCP-1 and IL-6 levels, the design of the cross-section does not permit the establishment of causality or prediction into the future of these mothers regarding such complications. It does so by laying down building blocks for subsequent, more extensive longitudinal studies.

## Conclusions

The outcomes indicate that women having gestational diabetes have a profile in terms of sociodemographics that is significantly different from that of controls, with added risk factors such as smoking and alcohol consumption and added obstetric complications such as hypertension, preeclampsia, and macrosomia. Furthermore, women with gestational diabetes were found to have lower levels of IL-6 and MCP-1, but not IL-18. Such results set the groundwork for future studies to further understand the effects these cytokine levels might cause in long-term complications, as their contribution speaks towards insulin resistance and uterine infections.
